# Capturing Objective Functional Measures Using Smartphone Inertial Sensors: Feasibility and Usability Study With Older Adults

**DOI:** 10.2196/72511

**Published:** 2025-09-22

**Authors:** Christian Kempton, Kate Ryan, Sophie Clohessy, Peter Grinbergs, Mark T Elliott

**Affiliations:** 1 WMG, University of Warwick, Coventry, United Kingdom; 2 EQL Ltd, Chorley, Lancashire, United Kingdom; 3 Centre for Food Policy, City St George’s, University of London, London, United Kingdom; 4 School of Sport, Exercise and Rehabilitation Sciences, University of Birmingham, Edgbaston, Birmingham, B15 2TT, United Kingdom, 44 121 414 2789

**Keywords:** physiotherapy, musculoskeletal, digital health, self-management, inertial sensors, smartphone, telerehabilitation

## Abstract

**Background:**

Digital platforms and smartphone apps have the potential to help patients with musculoskeletal conditions receive targeted interventions and physiotherapy support at home. As musculoskeletal conditions are much more prevalent in older adults, it is important to determine whether these technologies are accessible and acceptable to this demographic, who may possess lower levels of digital literacy compared to younger adults.

**Objective:**

The study aims to evaluate the feasibility and usability of completing functional assessments while recording the activity using smartphone inertial sensors in adults 60 years or older.

**Methods:**

Participants (N=21) were recruited from a range of community settings to complete a 4-week home-based trial, recording unsupervised sit-to-stand and single-leg balance activities at least once per week using their smartphone. We analyzed the data quality and adherence by number of assessments per week from the uploaded datasets. Feedback on usability was assessed using interviews and the System Usability Score.

**Results:**

Inductive content analysis was used to identify 5 top-level categories: app, device, task, time, and personal perception. The mean System Usability Scale score was 81.2 (SD 17.5). The proportion of valid data uploads was 63.8% (81/127) for single-leg balance and 93.5% (58/62) for sit-to-stand measures. Adherence was high, with no significant deviations in the mean number of sessions completed or duration between sessions.

**Conclusions:**

Smartphone-based monitoring of functional activities can facilitate unsupervised, remote assessments, thus reducing burden on physiotherapy services and increasing the ability to monitor progress objectively. Activities should be considered for complexity and, where necessary, increase in difficulty over time. App-based feedback is essential to inform users of the progress and adherence to the activities.

## Introduction

Nonadherence to self-managed physiotherapy programs is considered to be high [1,2]. This can lead to poor outcomes when patients do not receive adequate support to complete their prescribed physiotherapy for musculoskeletal conditions. Digital platforms and smartphone apps can help patients with musculoskeletal conditions receive targeted interventions and physiotherapy support within their own home [3]. Traditionally, patients would receive a paper handout explaining the exercises; however, they can now use digital tools to receive guidance via videos and set the level of difficulty themselves [4]. These platforms offer an opportunity to provide continuous guidance and support to patients self-managing their physiotherapy by allowing them to take control of their routine; receive detailed instruction on the exercises; and in some cases, have a direct channel to chat or send messages to a physiotherapist [5].

However, while these platforms provide ample resources for patients, there is still limited feedback to clinicians on both adherence to the prescribed program and, moreover, how the patient is progressing and improving in terms of physical function. Most feedback relies on patient-reported outcome measures (PROMs) and other self-reported questionnaires, providing only a subjective insight into the outcomes [6]. App-based platforms can provide further quantitative statistics indicating the level of interaction with the app and thus the exercise routines but do not provide the required objective measures from assessments or any changes in physical function and performance of the patient over time [7].

Modern smartphones are equipped with an array of sensors that are primarily focused on tracking the movement of the user. Recently, the sensors have been limited for measuring the daily step counts of the user, with some more advanced approaches using the sensors for falls [8]. However, with the advent of apps to support physiotherapy self-management, there is an opportunity to use these built-in sensors to provide effective objective measures of both patient adherence and progression in self-managed physiotherapy.

In this study, a method that uses the sensors available in all smartphones to track and measure the activities used by physiotherapists to assess the physical function of patients with musculoskeletal conditions was developed. A number of studies have investigated the viability of using smartphones’ inertial sensors for measuring functional activities such as sit-to-stand (StS). The results show that smartphone sensors are valid and reliable in capturing StS [9], timed up and go [10], and postural balance [11,12]. The smartphone sensors have been shown to be sensitive enough to differentiate StS activity in frail versus physically active older adults [13]. However, there is limited knowledge around the practicalities of older adults using smartphones to record their functional assessments in an unsupervised environment on a regular basis.

In this study, the feasibility and perceived usability of a smartphone app that allows the recording of two activities (StS and single-leg balance [SLB]) in the most likely user demographic of older adults (aged 60 years and older) were investigated. We evaluated the study outcomes in three ways: (1) engagement, evaluating whether participants completed the full program of assessments at least once per week over the 4-week period; (2) data quality, analyzing the resulting sensor data to examine the proportion of recordings that could be assessed by the algorithm; and (3) usability, detailed feedback from participants on usability using a standard questionnaire (System Usability Scale [SUS]) and a phone-based interview at the end of the trial.

## Methods

### Participants

Participants were recruited irrespective of whether they did or did not have any current musculoskeletal conditions. Similarly, we did not require participants to have any prior experience in using smartphone apps or other digital health platforms for managing musculoskeletal conditions or accessing services. This allowed us to gain a more generalized range of viewpoints from the trial. We used the following inclusion and exclusion criteria, which participants were screened against, before being onboarded into the study.

The following inclusion criteria were used:

- Adults aged 60 years or older
- Self-reported confirmation that they can walk >20 m without assistance
- Ability to move from sitting to standing without assistance
- Has access to Apple iPhone 6 or newer

The following exclusion criteria were used:

- Any current or previous knee, hip, or ankle injuries or conditions that could be exacerbated by repeated StS activities
- Any condition that may result in dizziness or loss of balance when standing

### Recruitment

The research team was recruited from a wide range of community organizations and venues, including community centers, local charity groups, libraries, supported-living accommodations, and religious centers such as churches and mosques. A total of 21 local organizations were contacted with the support of the West Midlands Clinical Research Network [14]. Paper and digital format posters were distributed in these locations through relevant contacts at each organization or via an in-person visit by a member of the research team.

Participants registered their interest by providing their contact details (email or postal address) via (1) an online form, (2) email, or (3) telephone message. The participant information leaflet and consent form were then sent to those who were registered. For organizations and community sites where there were multiple participants, we coordinated via the organization’s contact a suitable time to visit the site to onboard the group of participants. Individual participants were contacted directly to arrange a suitable date and location to attend the onboarding session, which was held at the university or a local community venue.

### Ethical Considerations

The study received ethical approval from the University of Warwick’s Biomedical and Scientific Research Ethics Committee (ref. 147.20‐21). Participants provided written consent prior to participation in the trial. All participants who engaged with the trial received a £20 (US $27) gift voucher for their participation. Participant data were pseudonymized using participant ID numbers assigned during the consenting process. A formal data sharing agreement was put in place between the researchers’ host institution and the commercial organization that produced the app.

### Procedure

**Figure 1.**
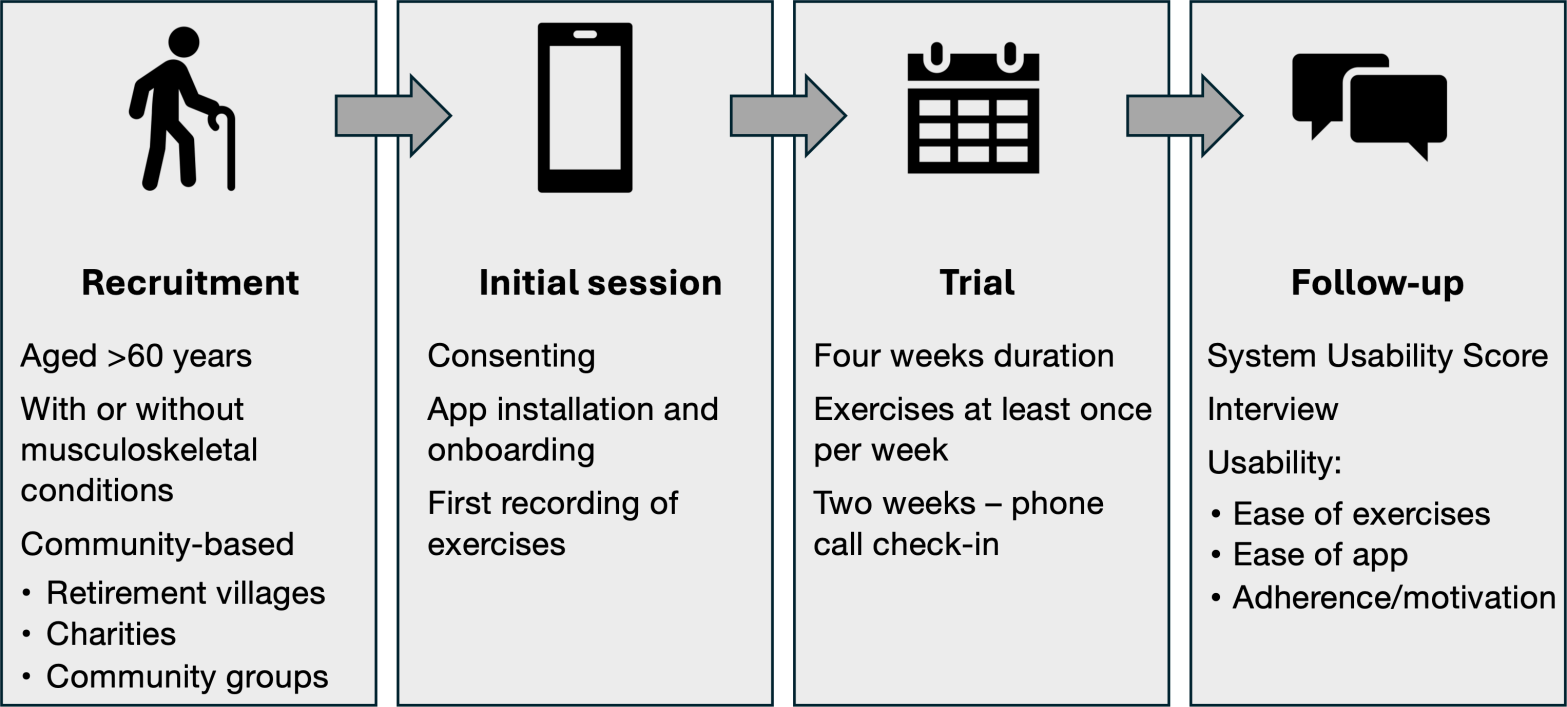
Key stages of the project.

The key stages of the project are shown in Figure 1. The trial began with an in-person onboarding session, where the participants were screened, consented, provided with guidance on installing and navigating the app, and demonstrated the functional activities. Initial recordings of the StS and SLB tasks were taken.

After the onboarding session, participants began the 4-week home-based trial. This involved using the app at least once a week to complete the two sets of assessments. Participants were able to complete the assessments at any time convenient to them during the week. No reminders were built into the app, so it was up to the participants to remember to complete the activities each week.

After the second week, the researcher contacted the participants over the phone to check that they were able to successfully complete the first 2 weeks of assessments. Any reported issues were subsequently resolved as necessary.

At the end of the trial period, the researcher arranged a phone interview for each individual participant to provide detailed feedback on their experiences during the trial. Interviews lasted approximately 30 minutes. A semistructured approach was taken using a list of guiding questions (provided in Multimedia Appendix 1). The researcher maintained detailed notes from the interview responses. Participants also completed the SUS [15] to provide a quantitative rating on the usability of the app.

### Software and Installation

The prototype app, named Phio Measures, was developed by EQL Ltd. The app worked by recording the StS and SLB activities using the inertial measurement units built into smartphones. Thus, the raw data recorded were the 3D measures of acceleration (using the accelerometer), angular velocity (gyroscope), and magnetic field (magnetometer), sampled at 100 Hz. Inertial data and relevant metadata (eg, trial type, user ID) were automatically uploaded to a secure server. The research team accessed the data via an online dashboard.

Offline algorithms were used to identify and record the time duration to complete 5 repetitions of StS. This used a peak detection method applied to the gyroscope data to identify the repetitions of movement in this activity, which could be visually checked for accuracy. For the SLB task, the algorithm measured the magnitude of sway (using the accelerometer data in anterior-posterior and medio-lateral directions), as well as the duration of completing the task before returning the foot to the floor. Significant foot contact was identified by a clear impact spike in the sway data, which was detected using a threshold approach. In addition, participants were asked to end the recording once both feet were back in contact with the ground. In this project, we did not assess the accuracy of the algorithm but rather determined the proportion of uploaded trials that the algorithms were able to successfully analyze (ie, completed analysis without error and correctly identified the start, end, and repetitions of the movements). In the case of SLB, the algorithm typically required >11 seconds of data to calculate the measures.

**Figure 2.**
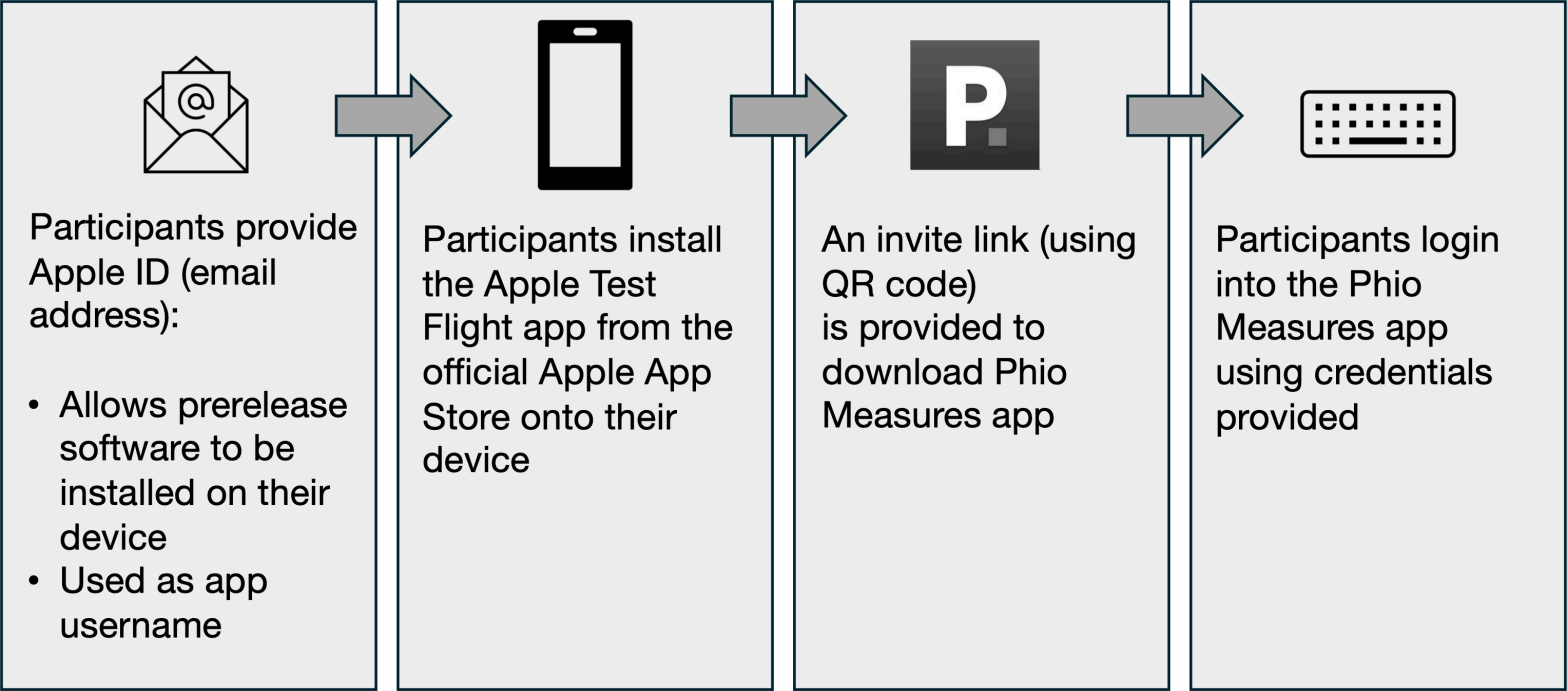
App installation process.

The app was only available to Apple iPhone users, and this was made clear in all the recruitment literature and communications. However, 1 participant had to be excluded due to having an iPhone that was too old to install the software. After completing the consent process, participants were assisted with installing the software onto their smartphones. As this was prototype software, it involved installing an additional Apple app, TestFlight, which allowed the installation of prerelease software onto the phone. The steps for installation are shown in Figure 2. Participants were able to delete the software from their device at the end of the trial or at any time during the trial (which was subsequently noted as a wish to withdraw from the trial).

### Functional Assessments

#### Overview

**Figure 3.**
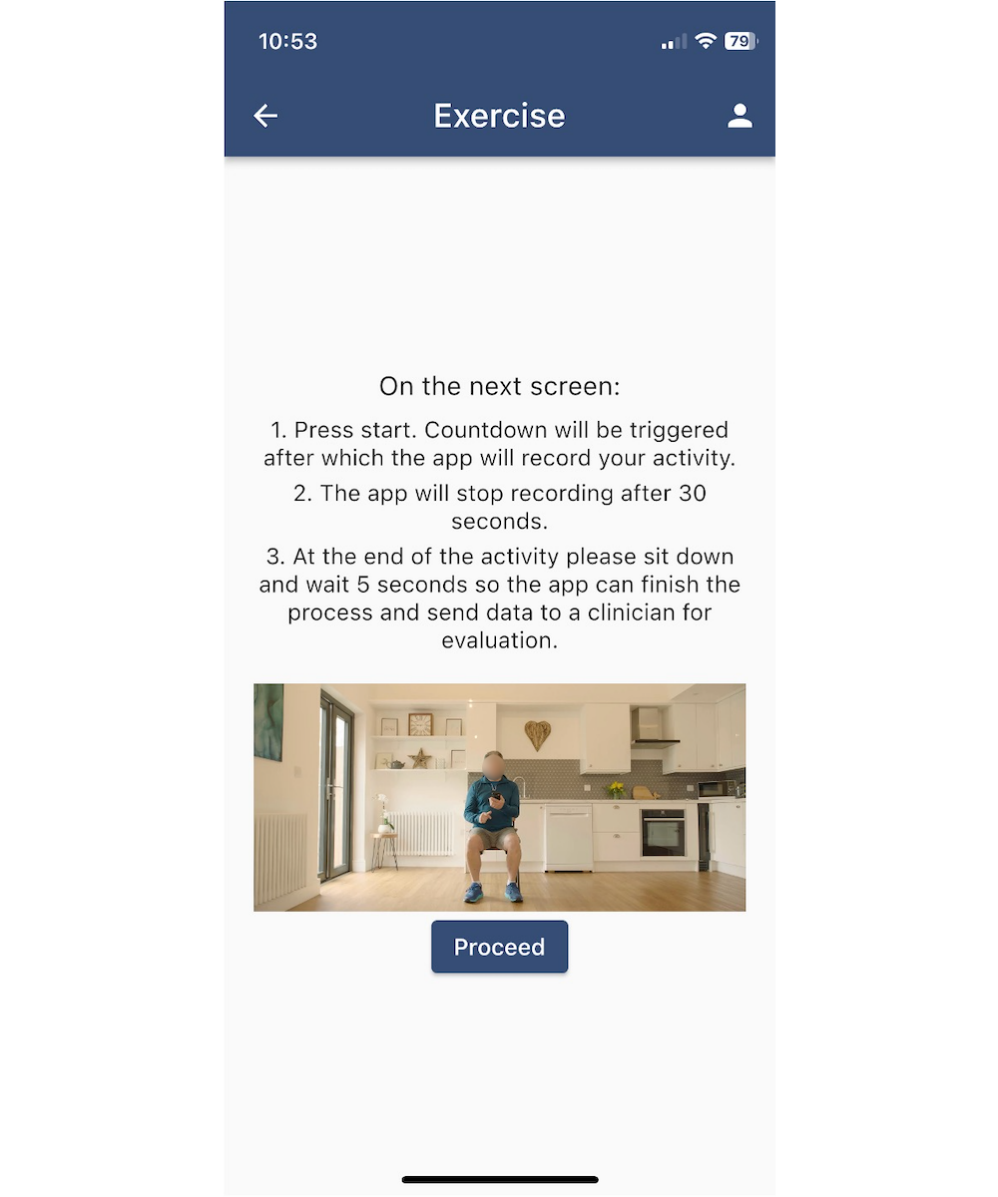
A screenshot of the app interface, showing a video and instructions of how participants record the sit-to-stand activity.

The trial involved completing 2 different types of functional activity on a weekly basis. A video was provided within the app to show the activity (Figure 3). At the onboarding session, the activity was demonstrated by the researcher, and an initial recording was taken. Each activity was completed with the participant holding their smartphone to the chest in portrait orientation (long axis vertical), with the inertial sensors recording movements. Participants were required to use a chair with fixed legs (no castors), a fixed seat, and no armrests (optional). The data from the smartphone sensors were uploaded automatically at the end of the recording to a secure online server.

The activities were completed as follows (in no set order).

#### Sit-to-Stand Task

The participant was instructed to sit in the chair, holding the smartphone in their hand. When comfortable, they pressed the start button on the app, which counted down from 5 (with audio). During that time, the participant raised the phone to their chest (around the area of the sternum) and held it firmly in place (with the screen of the phone facing into the chest). The phone was held in place with both hands, one over the other, across the phone. Participants remained sitting still with their backs straight until they heard the countdown was complete.

After the audio tone, the participant began to complete the standard StS protocol, moving from the sitting to the standing position while keeping their hands on the phone to hold it to the chest as they moved. Participants were instructed that once fully standing, they should avoid unnecessary pauses and aim for a continuous movement when returning to the seated position. They repeated the process until they had done 5 repetitions or heard the audio tone to stop (which occurred after 30 s).

Upon completion, they resumed the seated position, remaining still for 5 seconds. They were then able to move the phone and press “stop” before returning it to the chest and remaining still for another 5 seconds, after which the recording stopped.

#### Single Leg Balance Task

The participant began in a standing position, positioned behind the back of a chair, which could be used for support if balance was lost during the task. Holding the phone in their hand, they pressed the start button on the app when ready. The app counted down from 5 (with audio). During that time, the participant raised the phone to their chest (around the area of the sternum) and held it firmly in place (with the face of the phone facing into the chest). The phone was held in place with both hands, one over the other, across the phone (although participants could use one hand to help maintain a steady balance during the task, if required).

After the audio tone, participants lifted one leg (participants could choose which leg to raise first), with the foot fully off the floor and with the eyes remaining open. They remained in this position until either a 30-second period passed or they could no longer maintain balance and returned the foot to the ground. After this, they remained standing still for 5 seconds. They were then able to move the phone and press “stop” before returning it to the chest and remaining still for another 5 seconds, after which the recording stopped.

The same task was repeated for the opposite leg.

### Outcome Measures

The study focused on the usability of the app and the recording processed during the assessments. A mixed methods approach was used to capture smartphone measures, usability scores, and qualitative interview data.

The primary outcome measure was the qualitative results from the feedback interviews conducted after the trial period.

The secondary outcome measures included usability, assessed using SUS (0-100) during the posttrial interview; data quality, measured by the proportion of successful algorithmic assessments from unsupervised recordings taken during the trial; and trial adherence, evaluated by the number of recordings per participant and the time intervals between sessions [15].

### Analyses

A fair notes approach [16] was used to record the responses from the interviews, which were facilitated by a member of the research team (CK). An inductive content analysis was used to identify the key categories and concepts that were mentioned by participants during the interviews. These were grouped and synthesized to identify the positive and negative recurring concepts along with any subcategories that emerged from the discussions. Analyses of the interview notes were undertaken independently by author MTE and subsequently cross-checked by CK, who suggested amendments to categories and content allocation.

The mean and SD of SUS scores were calculated across participants. The resulting value was translated to a percentile level compared to standardized usability scores from historic studies [17]. Descriptive statistics were used on other secondary objective measures.

## Results

### Overview

**Figure 4.**
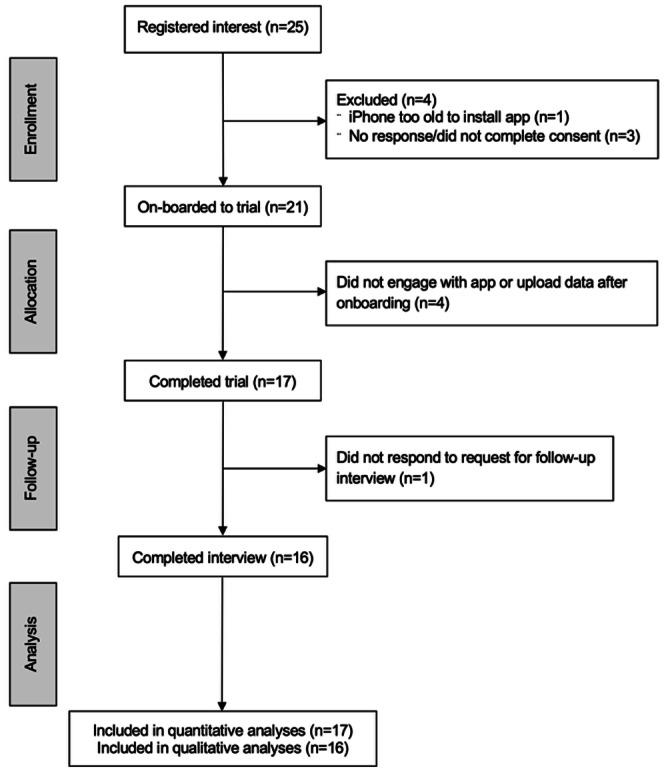
CONSORT (Consolidated Standards of Reporting Trials) flow diagram for the study.

A total of 25 individuals initially expressed interest in participating in the study (see Figure 4 for the CONSORT [Consolidated Standard of Reporting Trials] flow diagram for this study). Of these, 21 participants subsequently consented to take part in the study (female, n=15). One participant was unable to participate due to their Apple iPhone being too old to run the app; the remaining 3 did not respond to follow-up or complete a consent form. Finally, 17 participants completed the trial and provided valid data for analysis (female, n=12). The other 4 participants did not engage with the app, resulting in no data being uploaded (other than that collected during the initial onboarding session). One participant who completed the trial was unable to be contacted for the final interview.

### Proportion of Valid Trials

A total of 192 data recordings were uploaded by the 17 participants over the duration of the trial. Of these, 142 out of 192 (74%) were valid for subsequent analysis by the algorithm. There were a lower number of valid SLB measures (81/127, 63.8%) than StS measures (58/62, 93.5%). It should be noted that there were generally double the recordings of SLB (n=127) compared to StS (n=65) due to the recording of both left and right legs. Most of the invalid uploads were due to insufficient data samples recorded to perform an analysis, likely due to the participant placing their foot on the floor (recognized by a signal spike in the sensor data) after a very short duration and stopping the recording.

### Engagement

Participants were requested to complete both the StS activity and SLB for both legs at least once per week over the 4-week period of the trial. Participants, on the whole, adhered to the trial requirements (Table 1), completing a mean of 3.8 (SD 1.2) sessions for each activity, with just over a 7-day gap between sessions and a total duration between the first and the last sessions of just above the expected 21 days. Using 1-sample *t* tests against the target values, there were no significant deviations by participants from what was instructed (*P*>.05). Five participants were noted to have completed more sessions than requested (5‐6 in total) by completing multiple sessions in 1 week or continuing beyond the 4-week period. In contrast, 7 participants completed fewer than the requested number of sessions (2‐3 in total).

**Table 1.** Results indicating level of participant engagement and adherence to the 4-week trial period^a^.

	Target	Mean	SD	Min	Max
Trial duration, d^b^	21	23.1	14.3	2.0	47.0
Days between sessions	7	7.9	6.1	1.0	23.0
Number of sessions (sit-to-stand)	4	3.8	1.3	1.0	6.0
Number of sessions (single-leg balance)^c^	4	3.8	1.2	2.0	6.0

a Target represents expected values if the protocol was exactly followed.

b Number of days between first and last session.

c Recordings of left and right leg classed as one balance session.

### Objective Data Records

The overall mean time to complete the 5 repetitions of StS was 19.1 (SD 2.6) seconds. The overall mean time participants maintained SLB was 13.7 (SD 0.9) seconds. It was noted that the algorithms used were unable to calculate the sway measures when the duration of SLB was <11 seconds.

### System Usability Score

The mean SUS rating across all participants was 81.2 (SD 17.5; range 45-100). This is equivalent to being rated in the 90th percentile against other studies reporting the SUS score [17].

### Usability Feedback

**Figure 5.**
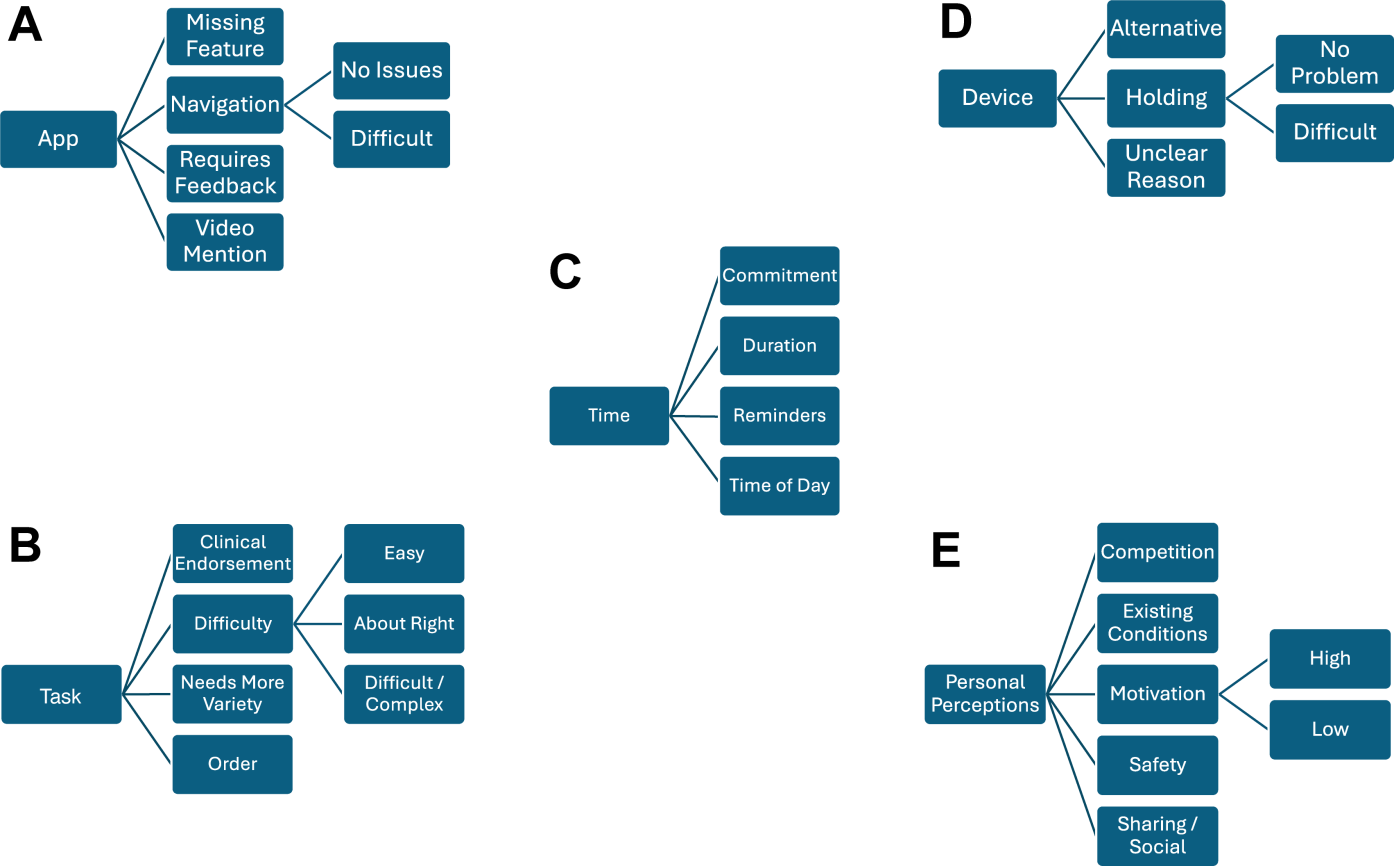
Schematics of the main themes identified as (A) app, (B) task, (C) time, (D) device, and (E) personal perceptions. Themes were identified using content analysis, with the resulting subcontexts and subthemes shown for each of the 5 top-level themes.

A total of 16 telephone interviews were undertaken with participants. Five top-level categories of discussion were extracted: app, task, device, time, and personal perceptions. These categories are a mix of those that emerged directly from the subject of the questions asked, as well as those that emerged indirectly through the discussion and have been identified in the analysis. Within the top-level categories, the recorded notes and quotations were further classified into sub-concepts, as shown in Figure 5. The following section discusses the context of each category and subcategory, providing representative quotes that were identified from participant interviews.

### App

#### Missing Features

Many participants were forthcoming in identifying missing features within the app and made recommendations on what future versions should include. These included reminders to complete the assessments, acknowledging that the data have been saved and uploaded, more buttons for navigating the app, an option to use voice activation to start and stop recordings, a progress bar during the assessments, and audio descriptions for the currently silent instruction videos.

#### Navigation

Ten participants reported that they found the app easy to use, with no problem navigating, due to the basic but simple interface (“Is old school style - less sophisticated & very simple, which…some might appreciate”). It was highlighted that if the number of functional assessments or activities were to increase, then a “facelift” would be required to make it more personalized. Of the participants mentioning issues with navigating, this included issues around logging in (“loss of interest initially…due to needing to log in every time”). There were also challenges around starting/stopping recordings, and because the phone was held to the body, they also remarked on being unable to see the screen during use (therefore possibly indicating a need for audio or vibrotactile indicators).

#### Feedback

It was clear that receiving feedback from the app is an important requirement, with comments highlighting that this was a missing feature. Participants were particularly keen to receive feedback on the assessments and progression over time (“Not motivated - no telling why doing exercises, not tailored, no reminders, not even a smiley face”). Furthermore, feedback on whether the activity and recording were successful was also deemed important (“I’d like feedback to say it’s definitely done. Would encourage me to do more and keep motivated”).

#### Video

The instructional videos embedded in the app were described as a “very useful visual guide.” It was highlighted that it would be better if they explained how they help with musculoskeletal conditions and why they are doing the activity.

### Task

#### Clinical Endorsement

Two participants mentioned it would be useful to have input from a physiotherapist as part of the program or to allow physiotherapy endorsement or association with the program.

#### Difficulty

Just over half of participants (n=9, 56.3%) reported the functional tasks to be “easy” or about the right level of difficulty. They highlighted the simplicity and consistency of the program and recognized that “anything you do is better than nothing.” The remainder had reported some difficulties, mostly related to SLB (“balance an issue,” “tiresome standing on one leg,” “[I] do wobble”). One specific issue related to StS was mentioned by a participant with current knee pain. The lack of feedback increased the perception that errors may have been made and concern that they were “cheating” when trying to steady themselves. A specific issue identified was that the 5-second countdown to the start of recording was not long enough to get “composure,” particularly if other more complicated assessments or activities are introduced.

#### Needs More Variety

The two functional assessments used during the trial (StS and SLB) were commonly identified as limited and that there should be more variety in the activities recorded. This included the option for more “vigorous” exercise to raise heart rate and “pressure/strength monitoring.” A “suite” of exercises or assessments was suggested (“Give choice of 3 a day out of a basket of 5…so it doesn’t get boring”) with the option of the physiotherapist being able to select the most relevant set for the patient. In addition, different levels of challenge were recommended including the use of gamification.

#### Task Order

Two participants mentioned the order of the functional assessments, generally selecting to do StS first.

### Device

#### Holding

Six participants specifically mentioned difficulty holding the phone during the tasks, describing it as “cumbersome” and “too challenging.” It was further thought that holding the device in the hand was affecting their balance. Additional difficulties faced while holding the device, due to conditions such as rheumatoid arthritis in the hand, were also highlighted. The remainder of the comments were positive, reporting no difficulties holding their device during the tasks, although one did forget to place it against their chest the first time they attempted it.

#### Alternatives

Alternatives to using the phone for recording activities were offered by participants during the interviews. These included smartwatches (eg, Apple Watch and Fitbit), tablets (eg, iPad), use of printed sheets for those who did not have access to a smartphone, and use of YouTube videos (for guidance only).

Suggestions of how to attach the phone were also made, including a “wearable chest strap” or “wear on the sleeve.”

#### Unclear Reason

Although only mentioned by one participant, an important point was highlighted that there was “no explanation why the phone is being held there.” They felt this diminished motivation to comply with requirements due to the lack of explanation.

### Time

#### Commitment

The convenience of the short time commitment required to do the assessments once per week was acknowledged, and that even when busy, it was possible to commit the time to complete the activities due to the short exercises (“Enjoyed it. Didn’t take much time, only minutes,” “10 mins is nothing out of the day”).

#### Duration

The duration of both the StS and SLB tasks was 30 seconds. Only one participant mentioned this seemed a long time (to maintain balance). Another participant suggested gradually increasing the duration of tasks to improve ability over time.

#### Reminders

There were 8 specific mentions about having reminders in the context of timing of the activities (eg, “Would use a reminder if doing seriously, i.e., if it was led by GP”, “…reminders…to do every 2-3 days”). However, a smaller minority of participants also mentioned that they did *not* require reminders and were able to remember (“No reminder needed. [Did exercises] whenever I had time.”), with one person highlighting that reminders may not be practical if the assessments were done at varied times across different days to fit in with other priorities.

#### Time of Day

It was commonly reported on how the activities fitted into daily routines; this included doing them in the periods “when most convenient or when least busy,” and more generally fitting it alongside other daily routines (“Usually around lunch, fits in after walking the dog,” “Normally after taking painkiller,” “Fit into own current exercises done frequently in the morning”).

### Personal Perceptions

The final theme captured any personal perceptions that completing the assessments had on participants or could have on participants, along with additional requirements identified for them to use this formally in the future.

#### Competition

Although one participant suggested gamification of the activities, when asked if there should be a competitive element to these activities (eg, use of leaderboards), there was no overall interest in adding these activities.

#### Existing Conditions

Participants with existing conditions mentioned how their condition impacted completing the tasks. In particular, a participant with dementia was unfamiliar with using a smartphone and needed substantial guidance. Cases of vertigo and instability in the right ankle also specifically impacted the SLB task.

#### Motivation

There were a small number of comments (n=6) relating to a lack of motivation to use the app or do the activities. The lack of feedback features led to statements that the app and activities were “dull and slow” and that the participant was “not motivated.” Another highlighted that they were motivated by the fact that it was researching into “treating ailments” but were not so happy that it was developed by a commercial organization. In contrast, there were 10 mentions of being positively motivated by the app and the activities. Simplicity, accessibility (“Can do at home in front room”), and short time commitments were mentioned as reasons for being motivated to engage with the activities.

#### Safety

When asked, all but one of the participants were positive that the activities were safe to do at home without clinical supervision. However, one person mentioned the SLB task did feel unsafe and was “too worrying” to complete alone.

#### Sharing or Social

Only one participant shared details of the activities with other members of the community, of whom “many have knee osteoarthritis,” while another discussed the trial with other “users” (based on the recruitment through community centers and supported living residences). Therefore, on the whole, there was limited evidence that participants shared their performance or experiences with their wider peer groups.

## Discussion

### Principal Findings

The aim of the digital platform trialed in this study was to be able to collect quantitative, objective measures of physical function when patients complete commonly used physiotherapy performance-based tests without clinical supervision. Our focus was to investigate the feasibility and usability of such a platform when used by older adults (aged 60 y or older), the group with the highest prevalence of musculoskeletal conditions [18]. The app was evaluated in three different ways: (1) engagement, evaluating whether participants completed the full program of assessments at least once per week over the 4-week period; (2) data quality, analyzing the resulting sensor data to examine the proportion of recordings that could be assessed by the algorithm; and (3) usability, detailed feedback from participants on usability using a standard questionnaire (SUS) and a phone-based interview at the end of the trial.

Our key findings were that overall, participants adhered to using the app at the minimum scheduled frequency over the duration of the trial. The quality of the data uploaded unsupervised by the participants during home-based completions of the activities was high for the StS task (93.5%), but much lower for the SLB task (63.8%), highlighting the challenges of completing a balance task by the participants. Despite its limited functionality as a prototype, the app scored highly on the SUS scale, likely due to the simplicity of the interface. However, this effectively provided a blank canvas for participants to recognize the desired features that were missing; they subsequently highlighted specifically the need for rapid feedback on their performance/outcome following completion of the tasks and the option for more activities to be made available to be chosen either by the individual or a medical professional (eg, physiotherapist, general practitioner). Short assessments or activities are essential and allowed participants to fit in alongside their daily activities. The proposed method of holding the phone to the chest while completing activities was considered feasible, but not ideal. Suggestions were put forward to improve this approach, including the use of a smartwatch; however, such devices are less prevalent in terms of ownership compared to smartphones, risking digital exclusion.

### Engagement and Adherence

Overall, there was a good level of engagement with the trial, with a total of 4 (19.1%) participants dropping out over the trial period. This contrasts with a meta-analysis study of mental health smartphone apps, which found an overall dropout rate of 26.2% (47.8% when accounting for publication bias) [19]. Our low dropout rate and high engagement are likely due to the short duration of the trial (4 wk). A longer-term program might result in a reduced sense of novelty and subsequently a significant dropout rate over time, without further intervention (eg, the use of incentives [20,21]). However, older adults are more likely to engage with remote digital health studies for a longer period than younger counterparts [22].

It was observed from the interview outcomes that the short, easy-to-complete activities were another driver of engagement, with participants able to fit them into their regular daily routines. While it may be considered that older adults have more leisure time than young adults to complete these activities, an Australian study found that 16% of inactive adults aged 65‐69 years reported not having enough time as the first or second highest barrier to exercise [23]. While the activities in this study are functional rather than cardiovascular-type exercises, it is important that their duration remains short. The Osteoarthritis Research Society International recommended performance-based tests for osteoarthritis [24] include StS (chair stand), along with a number of other simple activities, such as timed up and go, 6-minute walk, and stair-climb. Technically, all these activities could be recorded using standard smartphone sensors, but as reflected by our findings, each should be tested for its feasibility in unsupervised conditions to allow for unforeseen complexities in the task.

Our results further highlighted that only the StS task was practical for unsupervised monitoring using a smartphone. The low data quality of the SLB task was further reflected by the participants’ remarks, with some struggling to perform the balance task successfully. Participants also requested a variety of activities, which might involve more practical functional tasks such as a 10-m walk, timed up and go, or a stair climbing task [24].

Maintaining motivation to complete the activities is essential for longer term programs. While an increased range of exercises is one method of maintaining engagement, the other aspect highlighted by a substantial number of participants was to have feedback. The lack of feedback in the prototype was identified as a significant omission, with requests for both performance-based feedback (eg, time to complete 5 repetitions) and acknowledgments around the task (eg, task successfully completed, data uploaded). Performance-based feedback can give participants a sense of achievement, particularly if historical trends of performance are recorded [25]. However, it is noteworthy that when asked, no participants showed any desire to make these activities “competitive” (eg, using leader boards or comparing with friends).

### Data Quality

A significant concern in collecting data for unsupervised activities using devices such as smartphones is the reduction in data quality [26,27]. The activities need to be clearly defined and simple to execute with limited opportunity for alternative interpretation. Algorithms must have a level of flexibility to ensure there is some resilience against variation in the orientation of devices and varied sensor performance across smartphone devices (usually compared using step count variance [28-30]). In this study, the data analysis algorithms were implemented offline, after the sensor was uploaded to the server (thus removing the provision of feedback that many participants desired); near real-time analysis will be required for the app to be practical in the future, and this will require suitable detection of invalid recordings to avoid false feedback, particularly for clinical assessments. Data quality remained high, however, particularly for the StS task, where 93.5% of recordings were valid and able to be analyzed. Other studies have also confirmed smartphone sensors can provide a reliable and consistent method of capturing this activity [9,31]. The SLB was more concerning, with only 63.8% of recordings being usable. This reflects the challenge of the task, highlighted by some participants in the interviews, with one participant stating it also felt unsafe. Therefore, it is recommended that the StS activity is an activity that produces robust data for smartphone assessments while also being a useful functional assessment clinically [24]. On the other hand, the SLB task in its current form used in the trial is less practical for unsupervised, digitally recorded assessments. This could potentially be resolved by gradually increasing the difficulty of such a task. For example, rather than immediately requiring participants to complete a full 30 seconds from the start, the duration could be slowly increased over time, monitoring and adjusting the difficulty as users achieve goals.

### Usability

The mean SUS score provided by the participants after using the app for 4 weeks was somewhat surprisingly high. However, it has been noted that smartphone apps do typically score highly on the SUS, with a study investigating a range of apps reporting an average SUS score of 77.7 [32]. Our mean score of 81.2 is therefore in the upper range expected of apps. This is likely due to the simplicity of the interface we had in this prototype; the limited features and options implicitly made the app relatively easy to use. Adding more features (which participants strongly indicated were required) would therefore benefit from a co-design approach, such that potential end users would be involved in the design cycle to ensure usability is not lost as the complexity of the app increases.

### Limitations of the Study

There were some limitations emerging from this study. Unfortunately, due to information not collected on some participants and issues with the lead university not agreeing to licensing terms on the musculoskeletal questionnaire to be used, we gathered limited demographic information—particularly in terms of the overall age distribution of participants and level of function and mobility. However, in a related study in which some of the participants from this study also participated, it was reported that 80% of participants had some form of current musculoskeletal condition [26].

In terms of the app, it is likely that the prototype nature of the app meant its interface and lack of feedback had some impact on perceptions. However, this also had the benefit of being able to assess the core features required to facilitate the use of the smartphone to record these functional activities. Moreover, the process of using and holding the phone to complete the assessments without supervision was a key aspect of the study, and one that was achieved using the prototype app.

### Conclusions

It was found that it is feasible for people to use the built-in inertial sensors in a smartphone to record physiotherapy-related functions. However, activities should be limited to simple, stable activities such as an StS task, rather than activities that lead to instability, such as SLB. It is recommended that more exercises be evaluated from a clinical, patient, and data quality perspective to establish a portfolio of activities that patients can perform independently and be automatically monitored objectively via an app. Feedback is essential and therefore requires more study into how this can be optimized to achieve long-term adherence. Overall, smartphone-based monitoring of functional activities can facilitate unsupervised, remote assessments, thus reducing the burden on physiotherapy services and increasing the ability to monitor progress objectively.

## Supplementary material

10.2196/72511 Multimedia Appendix 1 Content analysis categories and subcontexts.

10.2196/72511 Multimedia Appendix 2 Quantitative and qualitative study data.

## Abbreviations

CONSORT: Consolidated Standard of Reporting Trials
PROM: patient-reported outcome measures
SLB: single-leg balance
StS: sit-to-stand
SUS: System Usability Scale
