# Supplementary material for: Capturing Objective Functional Measures Using Smartphone Inertial Sensors: Feasibility and Usability Study With Older Adults
Source: JMIR Rehabil Assist Technol. 2025 Sep 22;12:e72511. doi: 10.2196/72511 (PMC12453571; doi:10.2196/72511)
Supplement: Multimedia Appendix 1 [file rehab-v12-e72511-s001.docx]

**Post Trial Interview Questions**

**1.     How did you find the exercises themselves?** **Have you done other physio exercises/assessments that you think might be useful to capture?**

**1b. Is it the right level of difficulty. i.e. (Too easy/hard)**

**2.     Did holding the phone while doing the exercises add any extra complexities/issues**

**3.     How did you find starting and stopping the activity - was it clear.**

**4.     When did you do these activities - did you target a particular time of day/activity**

**4a.     What do you think is a good balance of frequency I.e. daily/weekly**

**4b.     How much time did you feel it took out of your day**

**5.     Did you always remember or set yourself a reminder**

**6.     How motivated were you to complete the exercises each week? Did this change at all over the course of the trial**

**7.     Did you feel confident that you were doing the activity correctly? was there enough guidance? what else would assist?**

**8.     Did you feel safe doing the activities? Was there someone else around?**

**9.     What type of feedback would you like - are you interested to know the change performance over time?**

**10.  Exercise order – did this matter to you?**

**11.  Did you share your performance with other people.**

**12. What other features would be useful in helping to manage - (final open question of what other features would they like to see if given the choice**
